# Experimental Study on the Coupling of Freeze-Thaw Cycle and Chloride Corrosion of Alkali Slag Cementitious Materials

**DOI:** 10.3390/polym17111474

**Published:** 2025-05-26

**Authors:** Jing Zhu, Zhiming Li, Ying Huang, Yuankai Li, Yapu Huang, Hao Min

**Affiliations:** 1 College of Civil Engineering and Architecture, Harbin University of Science and Technology, Harbin 150080, China; lizhiming0506@163.com (Z.L.); 13696334828@163.com (Y.L.); huangyf_@163.com (Y.H.); meehyy@foxmail.com (H.M.); 2 Department of Civil Engineering, North Dakota State University (NDSU), Fargo, ND 58102, USA

**Keywords:** alkali-activated-slag cementitious material, recycled rubber, freeze-thaw cycle, chloride corrosion, coupling test

## Abstract

Alkali-activated-slag cementitious material (AASCM) is distinguished by minimal energy consumption, reduced pollution, and superior mechanical properties; however, it is prone to issues such as susceptibility to cracking and inadequate frost resistance. To facilitate its application in cold region construction, research on AASCM modifications was conducted following freeze-thaw cycle and chloride ion corrosion coupling tests. The test results show that the AASCM made of recycled rubber and coal ash does not change much in shape or appearance after 100 freeze-thaw cycles, with a mass loss rate of less than 5% and a compressive strength loss rate of less than 25%. Furthermore, the AASCM containing recycled rubber, coal ash, and straw fiber demonstrates an effective resistance to freeze-thaw and chloride ion coupling, maintaining its appearance and shape without notable changes and exhibiting a mass loss rate of less than 25% following 100 such tests. Following 100 tests for freeze-thaw and chloride ion coupling, the appearance and morphology of AASCM exhibited no significant alterations, with a mass loss rate below 5% and a compressive strength loss rate under 25%; microscopic analysis revealed that the C-A-S-H gel maintained a relatively dense and stable structure. Adding recycled rubber to the AASCM matrix can slow the spread of cracks, make the material more flexible, and make it more resistant to frost. Straw fibers can stop cracks from getting bigger, and adding coal ash helps make more C-A-S-H gel, which improves the AASCM’s mechanical properties.

## 1. Introduction

The Peak Carbon Action Plan aims to expedite the low-carbon transition of cement and other binding materials by 2030 [1]. Alkali-activated-slag recycled material (AASCM) is a slag-based green low-carbon material characterized by low energy consumption, minimal pollution, and mechanical benefits such as rapid hardening and early strength development; however, it is deficient in crack and frost resistance [2]. The extreme cold and elevated chloride ion concentration from de-icing salt in the Northeast are the primary factors contributing to the degradation of concrete durability in that region. This paper conducts a performance test of AASCM in the Northeast region under the combined conditions of freeze-thaw cycles and chloride ion corrosion [3].

Kato et al. [4] discovered that the diffusion of chloride ions correlates with the concentration of chloride salt solution within cracks, leading to the proposal and development of a concrete cracking diffusion model to elucidate the relationship between crack width and the diffusion coefficient. Fang [5,6,7] investigated the impact of coal ash and MgO supplementation on the shrinkage of alkali-activated cementitious materials. The findings indicate that the shrinkage of alkali-activated cementitious materials is less than that of conventional silicate cement when the activator is water glass. The incorporation of coal ash diminished the shrinkage of alkali-activated cementitious materials. Qun et al. [6] performed tests on the mechanical properties and frost resistance of rubberized concrete. The test results indicate that rubber particles diminish the mechanical properties of concrete, yet they decrease the rate of compressive strength loss during freeze-thaw cycles and enhance frost resistance. Zhao Yiping [7] utilized the exciter for slag and coal ash, concluding that the mechanical properties of alkali-activated cementitious materials improved with higher alkali dosages, while the drying shrinkage was maximized at a slag to coal ash ratio of 7:3 [8,9,10].

This article introduces a type of environmentally friendly fiber—straw fiber. As a renewable resource, the application of straw fiber in AASCM helps reduce the dependence on traditional non-renewable resources and meets the requirements of sustainable development [11,12,13,14]. The straw fiber is hydrophilic and can promote the dispersion of alkaline activators, optimize the distribution of hydrated products, and thus enhance the durability and impermeability of AASCM materials [15]. In addition, straw fiber can also enhance the thermal insulation performance of AASCM, and its thermal insulation effect is better than traditional materials [16,17,18]. For example, the surface properties of straw fibers can be improved through biological pretreatment, thereby enhancing their compatibility with the AASCM matrix. Therefore, the application of straw fiber in AASCM not only contributes to environmental protection and resource conservation but also improves the overall performance of the composite material through appropriate processing methods [19,20,21].

The aforementioned studies by international scholars indicate that research on frost resistance and chloride corrosion performance predominantly targets cement-based concrete, whereas investigations into AASCM primarily concentrate on mechanical and shrinkage properties, with minimal attention given to freeze-thaw-chloride ion interactions. This paper presents experimental research on the interplay between freeze-thaw cycles and chloride corrosion in novel AASCM, achieved through the optimization of admixtures (straw fiber, recycled rubber, coal ash), with the objective of addressing a research gap and offering theoretical support for the application of AASCM in cold climates.

## 2. Overview of the Experiment

### 2.1. Materials

AASCM is an early-strength material. To apply this material on a large scale, it is necessary to reduce the hydration heat and microcracks generated by the intense early hydration reaction. Specifically, to optimize the proportion of AASCM for balanced mechanical and drying shrinkage properties, these different types of admixtures using different amounts were selected to be investigated in this study. The test’s raw materials comprise S105-grade acidic slag powder, I-grade low-calcium coal ash, 60-mesh (0.25 mm) recycled rubber, straw fiber, potassium water glass, and sodium hydroxide.

#### 2.1.1. Slag

The S105 grade slag used in this test is the industrial waste produced in the process of reducing iron by Tangshan Tielan Company (Tangshan, China). The S105 grade slag used in this test is the industrial waste produced in the process of reducing iron by Tangshan Tielan Company, with the alkalinity coefficient Mo = (MgO% + CaO%)/(SiO_2_% + Al_2_O_3_%) = 0.87, which is an acidic slag (Mo < 1 (Mo < 1 is acidic slag; Mo > 1 is alkaline slag). Its specific surface area is 550 m^2^ /kg, all parameters meet GB/T 18046-2017 “Granulated blast furnace slag powder for use in cement, mortar and concrete” [22]. The slag chemical composition is shown in Table The chemical composition of slag is shown in Table 1.

#### 2.1.2. Coal Ash

The quality grade of fly ash can be divided into three levels according to “Fly ash used in cement and concrete” (GB/T1596-2017) [23], i.e., level I, II, and III. Its specific technical indexes are shown in Table 2. In this paper, the specific surface area of 600 m^2^ /kg of Class I low-calcium fly ash is selected, which is supplied by Heilongjiang Shuangda Power Equipment Group Fly Ash Products Branch (Harbin, China).

#### 2.1.3. Recycled Rubber

Common crushing methods for waste tires are generally divided into cutting and grinding, low temperature grinding, etc., can be obtained through the crushing process of recycled rubber powder, rubber powder according to the different particle size is generally divided into rubber chips (2–10 mm), rubber particles (1–2 mm), and rubber powder (0.045–1 mm). In this paper, 60 mesh (0.25 mm) of recycled rubber powder.

#### 2.1.4. Straw Fiber

**Figure 1:**
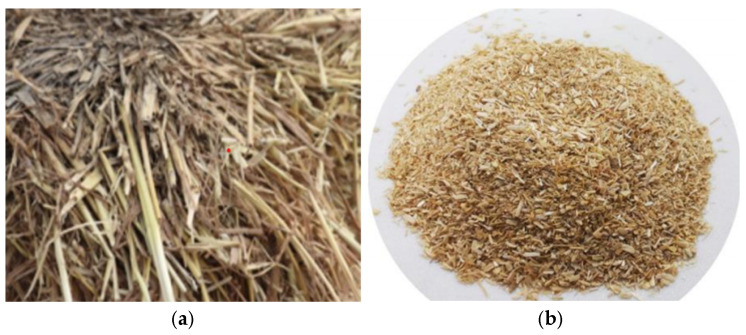
(**a**)straw fiber, (**b**)shredding.

The straw fiber is made of corn stalks from the northeast region. In order to prevent the fibers from agglomerating inside the body, the shearing treatment was carried out, and the straw fibers are shown in Figure 1.

#### 2.1.5. Standard Sand

The standard sand with SiO_2_ content > 96%, ignition loss ≤ 0.40%, clay content ≤ 0.20% was applied in this study and it was sourced from Xiamen ISO Standard Sand Co., Ltd. (Xiamen, China).

#### 2.1.6. Potassium Water Glass

Potassium water glass is provided by Xingtai Dayang Chemical Co., Ltd. (Xingtai, China) with the chemical molecular formula of K_2_O-n-SiO_2_-H_2_O, with an initial modulus of 2.78, a Baume degree of 46.3, a SiO_2_ content of 27.49%, a K_2_O content of 15.50% and the density was 1.434 g/mL.

#### 2.1.7. Sodium Hydroxide

Sodium hydroxide, NaOH (≥96.0% purity), a strongly alkaline compound, was used in this study, which was provided by Harbin Ligong Chemical Reagent Co., Ltd. (Harbin, China).

### 2.2. Mix Ratio Design

#### 2.2.1. Adjusting the Modulus of Potassium Silicate to 1.0

According to previous studies, the optimal modulus of potassium silicate used for the preparation of AASCM was 1.0 [24,25]. The original potassium silicate had a modulus of 2.78, which meant that the molar ratio in the solution was SiO_2_:K_2_O = 2.78:1. When the modulus was adjusted to 1.0, the molar ratio in the solution was SiO_2_:(K_2_O + NaOH) = 2.78:(1 + 1.78) [26,27]. Each gram of potassium silicate contained 0.155 g of K_2_O, which was converted to a molar amount of 0.0017 mol. To adjust the modulus to 1.0, the molar amount of NaOH added to each gram of potassium silicate should have been 0.006052 mol, which, when converted to mass, was 0.24208 g [28,29].

#### 2.2.2. Mix Ratio Design

In order to study the influence of different admixtures, 15 different mix designs were tested in this study, and the mix design numbers ranged from AAS-1 to AAS-15, the water gel ratio was 0.21. The material combinations required per cubic meter of AASCMS are shown in Table 3.

### 2.3. Pilot Program

#### 2.3.1. Compressive and Flexural Strength Test Methods

Add an appropriate amount of sodium hydroxide to potassium silicate with a modulus of 2.79 and stir evenly to make the modulus of potassium silicate 1.0. Pour slag and fly ash into a cement paste mixer (model: NJ-160A) according to the ratio and stir slowly for 1 min at a speed of 140 r/min to mix the powder evenly. Then, pour the potassium water glass and water into the blender, stir slowly for 2 min at a speed of 140 r/min, and then quickly stir for 2 min at a speed of 285 r/min.

Pour the mixed slurry into a 40 mm × 40 mm × 160 mm prismatic triple mold, ensuring that the slurry covers the mold as much as possible to prevent shortages in the size of the test block due to leakage during vibration, which can lead to errors in flexural and compressive strength. After vibrating on the vibrating table for 120 times, it is placed in a curing box with a standard temperature of 20 °C ± 2 °C and a relative humidity of 95% for curing. After 1 day of curing, it is demoulded.

**Figure 2:**
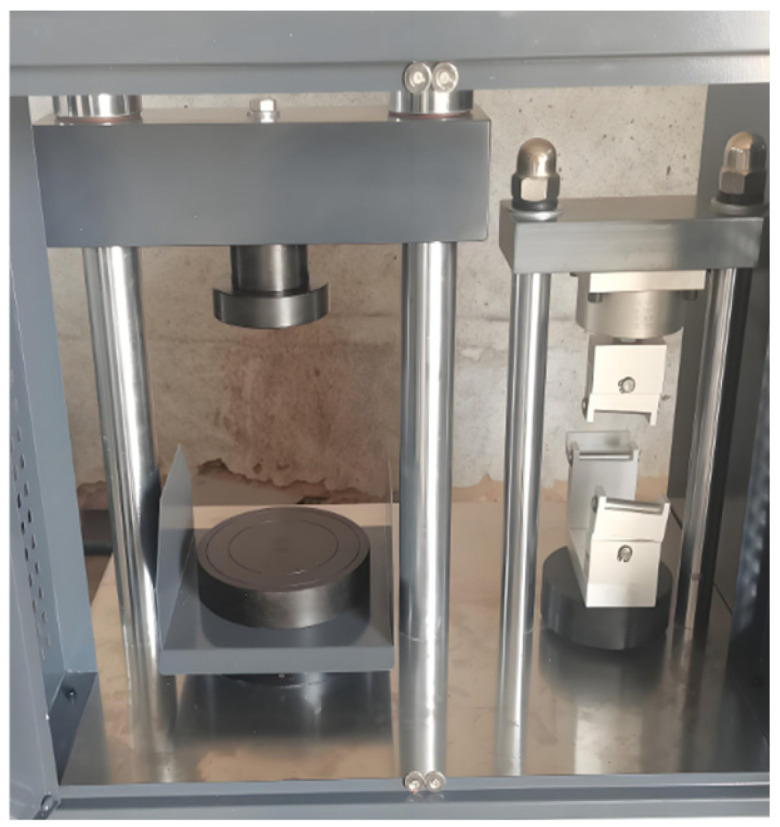
Compressive strength testing machine.

The specimens were cured for 3, 7 and 28 days and tested using the HYE-300B cement flexural and compressive constant stress testing machine, which manufactured by Quanzhou KeShuo Instrument Co., Ltd. (Quanzhou China), as shown in Figure 2, following testing standard JGJ/T70-2009 “Standard for test method of performance on building mortar” [30]. The flexural strength was tested by applying a load uniformly at a rate of 50 N/s until failure. After the flexural strength test was completed, the specimens were cut into two pieces. Then, the compressive strength of the intact specimens without any other damage was tested at a loading rate of 2400 N/s. The arithmetic mean of the measured values of three specimens was used as the compressive strength of the mortar cubic specimens for the test group.

#### 2.3.2. Freeze-Thaw Cycle Test Methods

**Figure 3:**
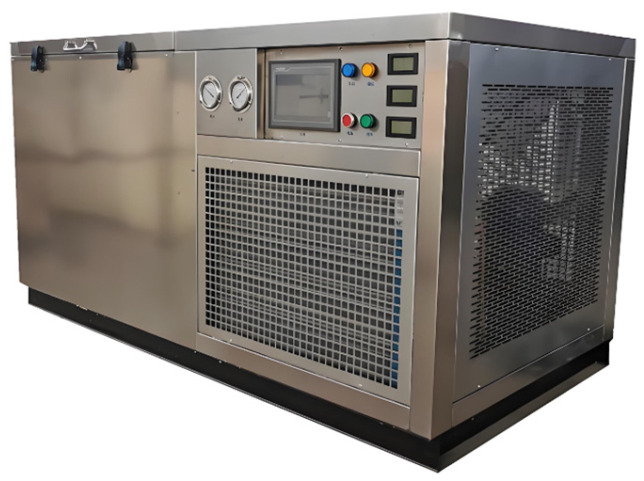
Fully automatic low temperature freeze-thaw tester.

In accordance with the Standard for Long-term Performance and Durability Test Methods of Ordinary Concrete (GB/T 50082-2009) [31], a freeze-thaw cycle test was conducted on 100 mm × 100 mm × 100 mm AASCM cube specimens utilizing the slow-freezing technique. Establish the freeze-thaw cycle with a freezing temperature of −18 ± 2 °C and a melting temperature of 18 ± 2 °C; cease testing upon reaching 100 cycles. The freeze-thaw testing apparatus is depicted in Figure 3 [30,31]. The rate of strength loss was determined using Equation (1):

(1) $$∆P=\frac{P_{0}-P_{C}}{P_{C}}\times 100\%$$

where ∆*P* denotes the percentage of strength loss after n freeze-thaw cycles; *P*₀ represents the initial average compressive strength (MPa) prior to the cycle; and *P*_c_ represents the mean compressive strength (MPa).

The rate of mass loss is calculated according to Equation (2):

(2) $$∆m_{ni}=\frac{m_{0i}-m_{ni}}{m_{0i}}\times 100\%$$

where ∆*m*_n*i*_ represents the mass loss rate (%) following n freeze-thaw cycles; *m*_0*i*_ denotes the average initial mass (g) prior to cycling; and *m*_n*i*_ indicates the average mass (g) subsequent to cycling.

#### 2.3.3. Chloride Ion Corrosion Test Methods

The chloride corrosion test was conducted through prolonged immersion, utilizing prismatic specimens measuring 40 mm × 40 mm × 160 mm.

(1) Measure the mass of NaCl solid at 5% concentration and the mass of warm water individually, combine them thoroughly, and gradually introduce the mixture into the immersion tank.
(2) Position the test specimens in a 5% NaCl solution after 28 days of maintenance for a long-term immersion test, ensuring that the liquid level does not exceed 20 mm above the top of the specimens, and cover the immersion container with a film.
(3) Extract a portion of the specimen every 30 days, eliminate surface moisture, and conduct analyses for chloride ion concentration and compressive strength. To maintain a consistent solution concentration, replace the NaCl solution every 30 days.

#### 2.3.4. Freeze-Thaw-Chloride Ion Coupling Test Methods

The freeze-thaw-chloride ion coupling test and the freeze-thaw cycle test method are identical. The test block must be maintained for over 28 days, followed by immersion in a 5% NaCl solution for 4 days. After removal, the test block should be dried to eliminate surface moisture and weighed. The water will be substituted with a 5% NaCl solution to conduct the freeze-thaw cycle test for 0, 25, 50, 75, and 100 cycles. After every 25 cycles, a portion of the test blocks was extracted, and their mass and compressive strength were assessed [32,33,34].

#### 2.3.5. Microanalysis Test Methods

**Figure 4:**
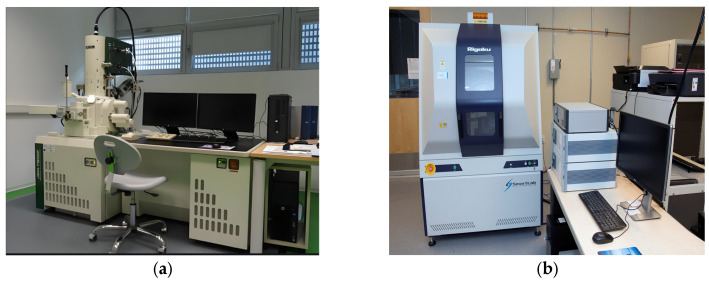
Diagram of microscopic test equipment. (**a**) Scanning Electron Microscope, (**b**) X-ray diffractometer.

(1) X-ray Diffractometer (XRD): XRD tests were performed on the samples from the specimens using D8-Advance from Bruker Corporation (Billerica, MA, USA). The X-ray diffractometer depicted in Figure 4b was utilized; the test block was crushed and subsequently ground into a powder using a mortar and pestle. The powder, which passed through a 0.075 mm sieve, was then placed in a beaker and dried in a drying oven at 40 °C until a constant weight was achieved. The physical phase was analyzed using X-ray diffraction (XRD) with a measurement precision of ≤0.010 and a scanning range of 10° to 90°.
(2) Scanning Electron Microscope (SEM): SEM analyses were performed on the specimens using SUPERTM 55 from Carl Zeiss AG (Oberkochen, Germany). In scanning electron microscopy, as depicted in Figure 4a, the center of the fractured section of the 28-day compressive test block is immersed in anhydrous ethanol to halt hydration for 7 days. Subsequently, it is removed and dried in a 60 °C oven until a constant weight is achieved. After drying, the sample is affixed to a tray for gold sputtering, followed by examination using scanning electron microscopy.

## 3. Results and Discussions

### 3.1. AASCM Freeze-Thaw Cycle Test

To assess the impact of admixtures on the frost resistance of AASCM and to explore optimal material composition and proportions, freeze-thaw cycle tests were performed on AAS-1, AAS-3, AAS-5, AAS-8, and AAS-10 and compared with C30 concrete to elucidate the principles of frost resistance. Consequently, the optimized ratio was determined for the freeze-thaw-chloride ion coupling test.

#### 3.1.1. Appearance and Morphological Changes of Specimens After Freeze-Thaw Cycle

**Figure 5:**
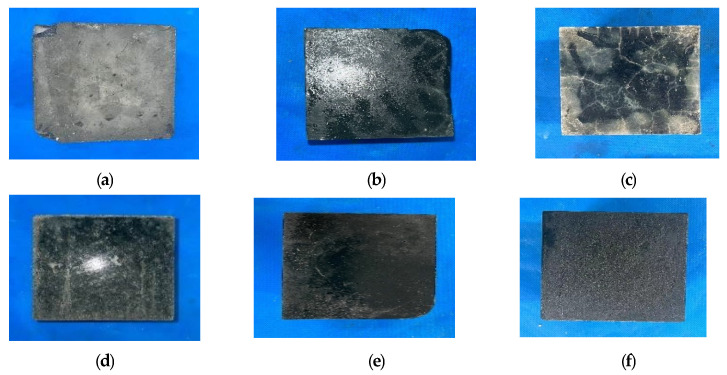
Appearance and morphology after 100 freeze-thaw cycles. (**a**) C30, (**b**) AAS-1, (**c**) AAS-3, (**d**) AAS-5, (**e**) AAS-8, (**f**) AAS-10.

Figure 5 illustrates the condition of each group of proportioning blocks following 100 freeze-thaw cycles. As can be seen from the figure, after 100 freeze-thaw cycles, the surface of the ordinary C30 concrete test block is severely eroded and the freeze-thaw damage is aggravated. After 100 freeze-thaw cycles, the surface produces a large number of cracks, pits, etc. However, there is no obvious freeze-thaw damage, indicating that the addition of recycled rubber directly affects the appearance damage of AASCM.

The freeze-thaw cycle test of each proportioning block indicates that the incorporation of recycled rubber directly influences the manifestation of damage in AASCM.

#### 3.1.2. Rate of Quality Loss

**Figure 6:**
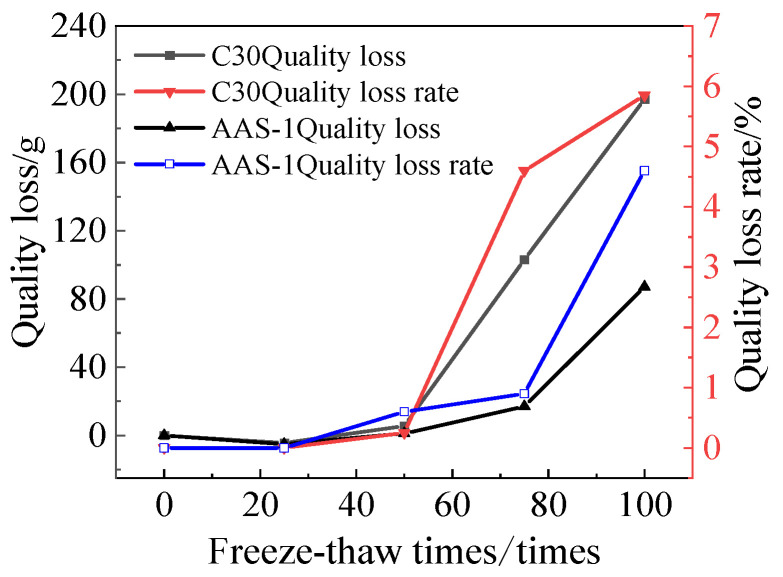
C30 vs. AAS-1 compressive strength loss and compressive strength-loss rate.

Figure 6 illustrates the mass loss and mass loss rate of C30 concrete and AAS-1. The mass loss rate of AASCM is significantly lower than that of conventional concrete, recorded at 5.85% after 100 freeze-thaw cycles.

**Figure 7:**
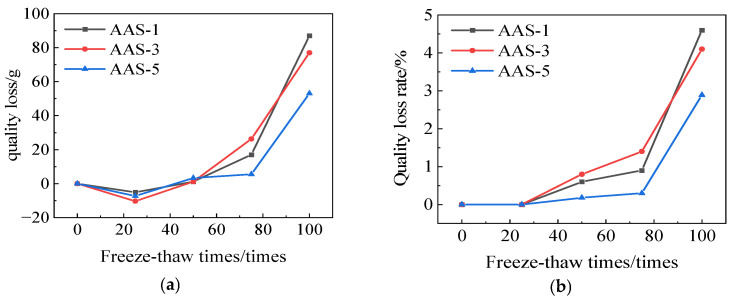
AAS-1, AAS-3, and AAS-5 compressive strength loss and compressive strength loss rate. (**a**) Loss of quality, (**b**) Rate of loss of quality.

Figure 7 illustrates the mass loss and mass-loss rate of AAS-1, AAS-3, and AAS-5. The mass loss rate of AAS-5 is 2.89%, while the mass loss rate of AASCM with 5% recycled rubber doping is approximately 2% lower than that of AASCM without doping. The incorporation of recycled rubber enhances the frost resistance of AASCM, surpassing the efficacy of coal ash alone.

**Figure 8:**
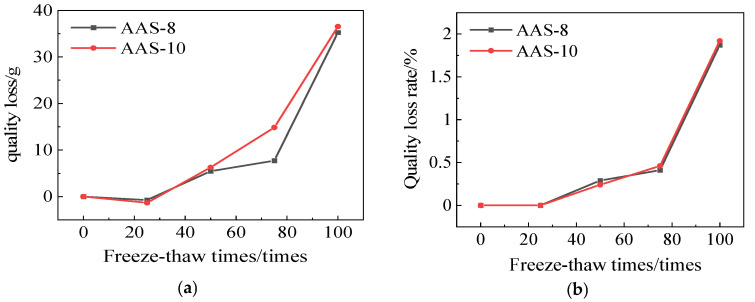
AAS-8 and AAS-10 compressive strength loss and compressive strength loss rate. (**a**) Loss of quality, (**b**) Rate of loss of quality.

Figure 8 illustrates the mass loss and mass loss rate of AAS-8 and AAS-10. The mass loss rates of AAS-8 and AAS-10 are 1.87% and 1.92%, respectively. The mass loss rate of AASCM, following the compounding of coal ash and recycled rubber, is lower than that of single compounding, and it exhibits superior freezing resistance.

#### 3.1.3. Rate of Loss of Compressive Strength

**Figure 9:**
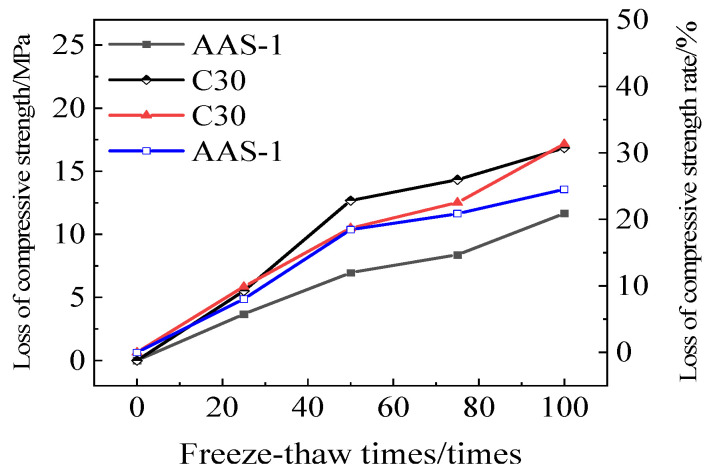
C30 and AAS-1 compressive strength loss and compressive strength loss rate.

Figure 9 illustrates the reduction in compressive strength and the rate of compressive strength loss for C30 concrete and AAS-1. The compressive strength loss of normal concrete after 100 freeze-thaw cycles is greater, at 31.34%, compared to that of AASCM.

**Figure 10:**
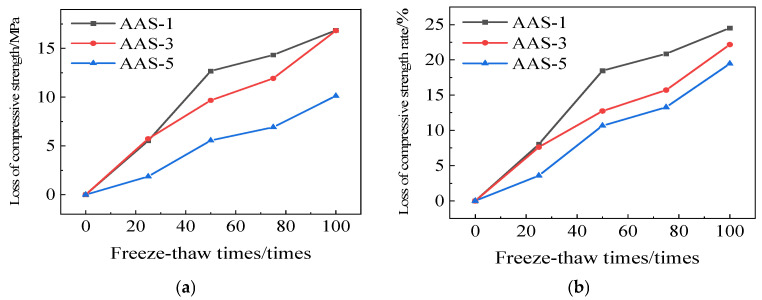
AAS-1, AAS-3, and AAS-5 compressive strength loss and compressive strength loss rate. (**a**) Loss of compressive strength, (**b**) Rate of loss of compressive strength.

Figure 10 illustrates the compressive strength reduction of AAS-1, AAS-3, and AAS-5, along with their respective rates of compressive strength loss. The rate at which AAS-5 loses its compressive strength is 19.46%, which is a big drop compared to AAS-1. Adding recycled rubber exclusively makes AASCM much more resistant to frost. The primary reason is that recycled rubber is an elastic material, which provides cushioning in the specimen, mitigates internal stress effects, and restricts the propagation of minor cracks in the frozen specimen.

**Figure 11:**
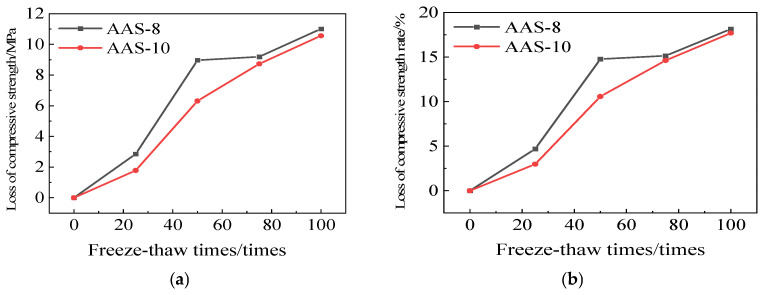
AAS-1, AAS-3, and AAS-5 compressive strength loss and compressive strength loss rate. (**a**) Loss of compressive strength, (**b**) Rate of loss of compressive strength.

Figure 11 illustrates the reduction in compressive strength and the rate of decline in compressive strength for AAS-8 and AAS-10. The compressive strength loss rates of AAS-8 and AAS-10 are 18.14% and 17.69%, respectively, after 100 freeze-thaw cycles. Furthermore, the compressive strength loss rate of AASCM, when combined with coal ash and recycled rubber, is lower than that of its individual components, indicating a more pronounced effect of this combination on the frost resistance of AASCM.

### 3.2. AASCM Chloride Corrosion Test

In order to determine the effect of admixtures on the chloride corrosion resistance of AASCM and to explore better material compositions and proportions, chloride corrosion tests were conducted on AAS-1, AAS-3, AAS-12, AAS-13, and AAS-14, which were compared with C30 concrete to analyze the principles of chloride corrosion resistance. Based on this analysis, the optimized proportion was selected for the test of freeze-thaw and chloride ion coupling.

**Figure 12:**
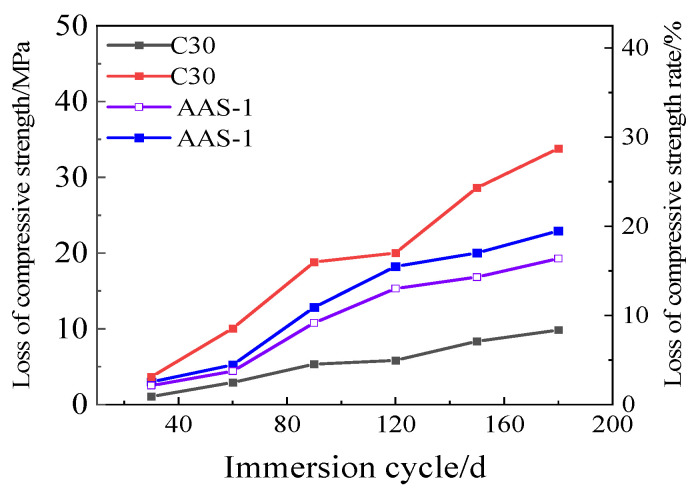
C30 and AAS-1 compressive strength loss and compressive strength loss rate.

Figure 12 shows the loss of compressive strength and the rate of loss of compressive strength for C30 and AAS-1. After 180 days of immersion, it is evident that ordinary silicate cement exhibits a higher loss of compressive strength and a higher rate of loss of compressive strength compared to AASCM. The compressive strength loss and the compressive strength loss rate of AAS-1 and AAS-3 are illustrated in Figure 12. It is clear that AASCM containing coal ash alone experiences a higher compressive strength loss but a lower compressive strength loss rate than AASCM without external admixture.

**Figure 13:**
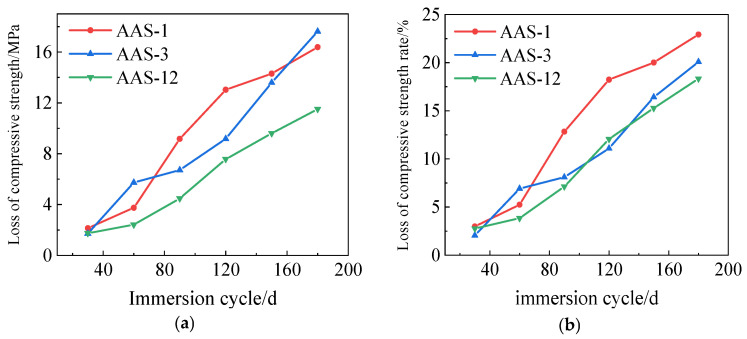
AAS-1, AAS-3, and AAS-12 compressive strength loss and compressive strength loss rate. (**a**) Loss of compressive strength, (**b**) Rate of loss of compressive strength.

The compressive strength loss and compressive strength loss rate for AAS-1, AAS-3, and AAS-12 are presented in Figure 13. The data shows that AASCM with only straw fiber has a much lower rate of compressive strength loss than AASCM without any other materials added and AASCM with only coal ash.

**Figure 14:**
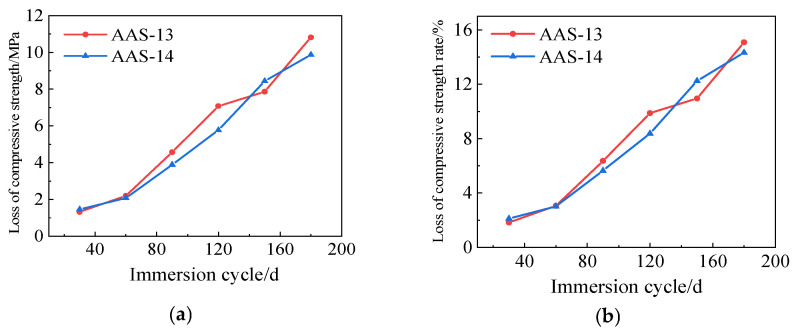
AAS-13 and AAS-14 compressive strength loss and compressive strength loss rate. (**a**) Loss of compressive strength, (**b**) Rate of loss of compressive strength.

The compressive strength loss and compressive strength loss rate for AAS-13 and AAS-14 are shown in Figure 14. The results reveal that the compressive strength loss and compressive strength loss rate for AASCM with compounded coal ash and straw fiber were significantly reduced. The compressive strength loss rate for AAS-13 was 15.09%, while for AAS-14 it was 14.33%. Both AAS-13 and AAS-14 exhibit a compressive strength loss rate that is approximately 3% lower than that of AASCM with straw fiber alone.

The results show that AASCM is better at removing chloride ions when 20% coal ash and 1% straw fiber are added together.

### 3.3. AASCM Freeze-Thaw-Chloride Corrosion Coupling Test

In practical engineering, damage from freeze-thaw cycles and chloride ion corrosion typically occurs simultaneously. So, tests that look at how freeze-thaw cycles and chloride ion corrosion affect AASCM are useful for engineering research that can be used in the real world. The control groups for the coupling test included C30 concrete, AAS-1, AAS-3, AAS-10, AAS-14, and AAS-15. When C30 concrete and AASCM with different ratios were damaged by freeze-thaw and chloride ion coupling, they were looked at in terms of how they changed in shape and appearance, how fast they lost mass, and how fast they lost their compressive strength.

#### 3.3.1. Appearance Changes of AASCM and C30 Concrete Specimens After Freeze-Thaw Coupling with Chloride Ions

**Figure 15:**
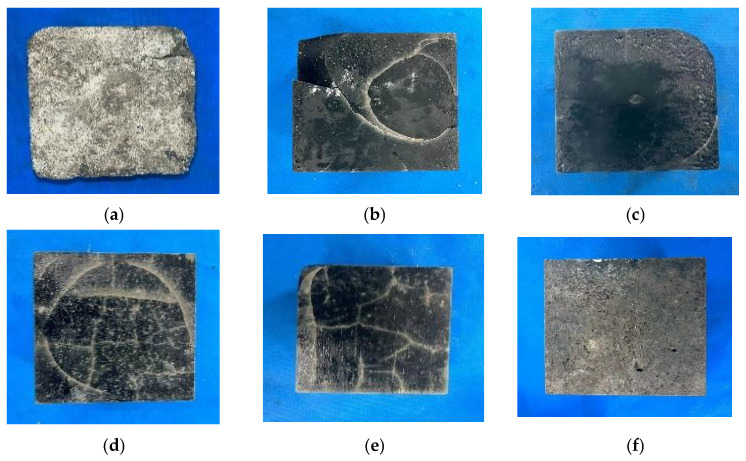
Appearance and morphology after 100 cycles of freeze-thaw and chloride ion coupling action. (**a**) C30, (**b**) AAS-1, (**c**) AAS-3, (**d**) AAS-10, (**e**) AAS-14, (**f**) AAS-15.

The appearance and morphology of the test block after 100 cycles of freeze-thaw-chloride coupling are shown in Figure 15. As shown in the figure, after 100 cycles of freeze-thaw-chloride coupling, the surface and interior of ordinary C30 concrete were severely eroded, with cracks and pits appearing on the surface of AAS-1 and AAS-3, and missing corners appearing on the edges of the test blocks. However, no obvious damage was observed on AAS-8, while AAS-14 had large surface cracks and fissures. After 100 cycles of freeze-thaw-chloride coupling, there is no obvious appearance damage in AAS-15, indicating that the combined use of fly ash, 60-mesh recycled rubber, and straw fiber can reduce the damage caused by freeze-thaw-chloride coupling.

#### 3.3.2. Rate of Quality Loss

**Figure 16:**
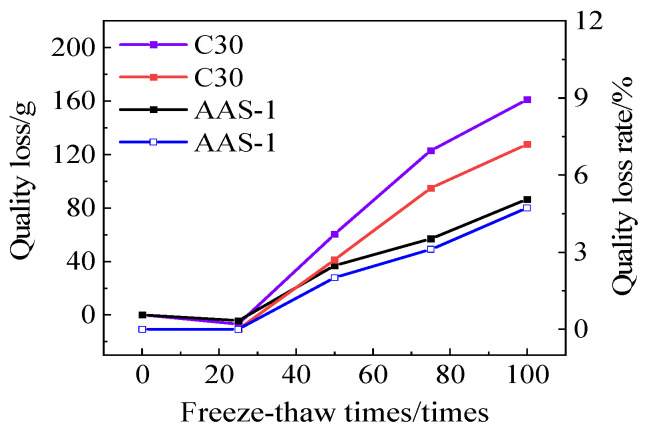
C30 and AAS-1compressive strength loss and compressive strength loss rate.

Figure 16 depicts the correlation between mass loss and mass loss rate for C30 and AAS-1. Following 100 cycles of freeze-thaw-chloride ion interaction, the mass loss and mass loss rate of C30 concrete surpass those of AASCM.

**Figure 17:**
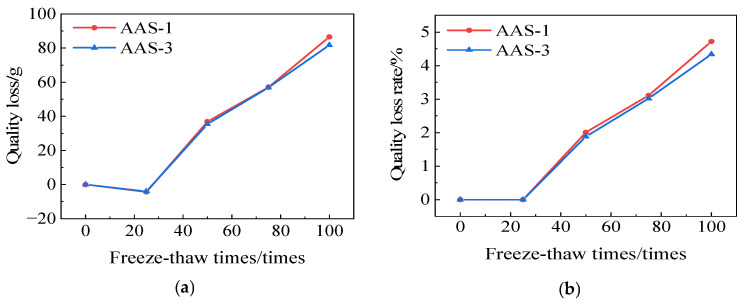
AAS-1 and AAS-3 compressive strength loss and compressive strength loss rate. (**a**) Loss of quality, (**b**) Rate of loss of quality.

Figure 17 illustrates the mass variations of AAS-1 and AAS-3. The mass loss of AASCM combined with coal ash and recycled rubber is 44.34 g, resulting in a mass loss rate of 2.39%; whereas the mass loss of AASCM combined with coal ash and straw fiber is 58.15 g, yielding a mass loss rate of 3.15%. Consequently, freeze-thaw damage predominated in the freeze-thaw-chloride ion coupling detrimental effect, and the regenerated rubber significantly enhanced the anti-freeze-thaw-chloride ion coupling efficacy of AASCM.

**Figure 18:**
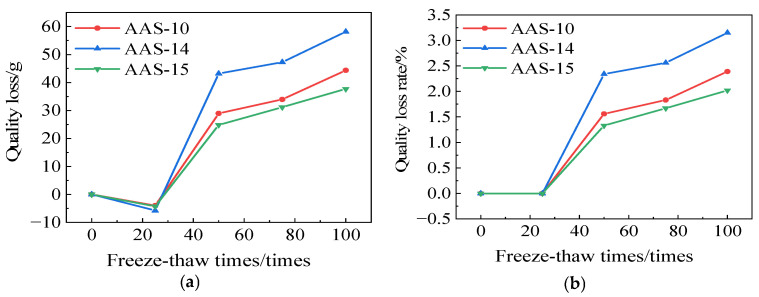
AAS-10, AAS-14, and AAS-15 compressive strength loss and compressive strength loss rate. (**a**) Loss of quality, (**b**) Rate of loss of quality.

Figure 18 illustrates the mass variations of AAS-10, AAS-14, and AAS-15. The mass loss observed in AASCM was 37.70 g, which resulted in a mass loss rate of 2.02% after incorporating coal ash, recycled rubber, and straw fiber. A notable reduction in both mass loss and mass loss rate is evident when compared to AAS-10 and AAS-14.

#### 3.3.3. Rate of Loss of Compressive Strength

**Figure 19:**
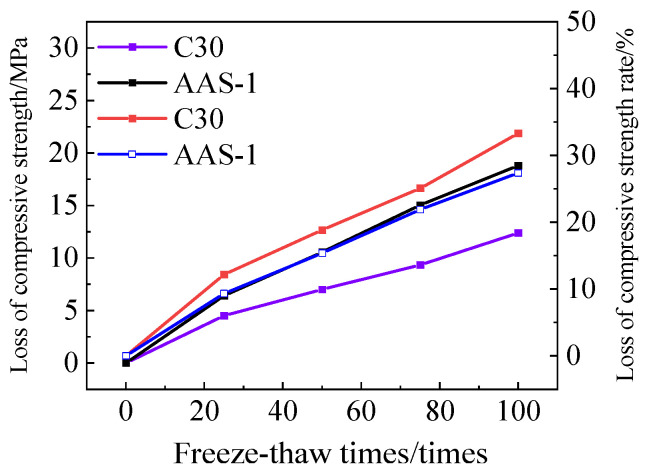
C30 and AAS-1compressive strength loss and compressive strength loss rate.

Figure 19 illustrates the loss of compressive strength and the rate of compressive strength loss for C30 concrete and AAS-1. After 100 cycles of freeze-thaw-chloride ion interaction, the compressive strength loss rate of ordinary C30 concrete is 33.28%, whereas the compressive strength loss rate of AASCM without external admixture is 27.36%. Standard C30 concrete is inferior to AASCM in its resistance to freeze-thaw-chloride ion interactions.

**Figure 20:**
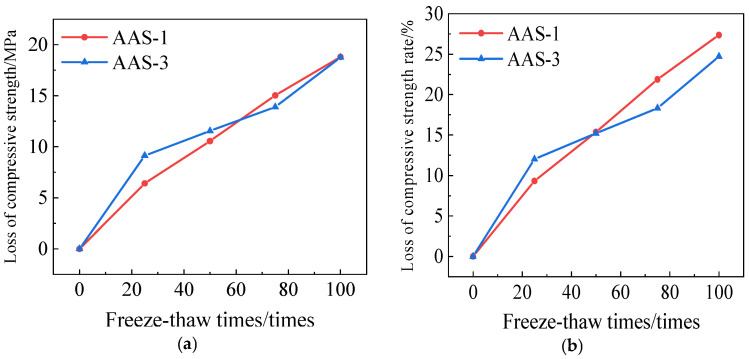
AAS-1 and AAS-3compressive strength loss and compressive strength loss rate. (**a**) Loss of compressive strength, (**b**) Rate of loss of compressive strength.

Figure 20 illustrates the variations in compressive strength of AAS-1 and AAS-3. After 100 cycles of freeze-thaw-chloride ion coupling, the compressive strength loss of AASCM without an external dopant and AASCM with a single dopant of coal ash is comparable; however, the rate of compressive strength loss in AASCM with coal ash is marginally lower than that of AASCM without an external dopant. AASCM with coal ash is marginally more effective than AASCM without external admixture in resisting freeze-thaw-chloride ion action.

**Figure 21:**
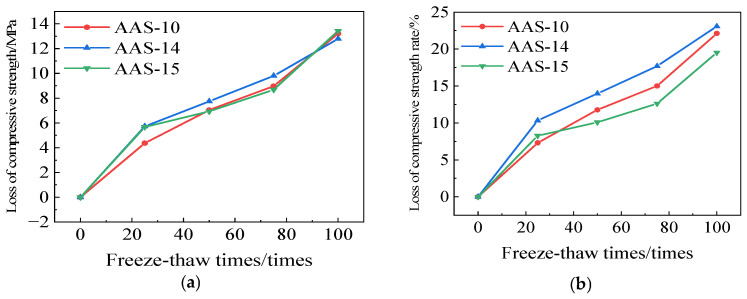
AAS-10, AAS-14, and AAS-15 compressive strength loss and compressive strength loss rate. (**a**) Loss of compressive strength, (**b**) Rate of loss of compressive strength.

Figure 21 illustrates the variations in compressive strength of AAS-10, AAS-14, and AAS-15. After 100 cycles of freeze-thaw-chloride ion exposure, the compressive strength loss of AASCM incorporating coal ash, recycled rubber, and straw fiber was 13.46 MPa. This loss was marginally greater than that of AAS-10 and AAS-14; however, the rate of compressive strength reduction was lower than that of both. Consequently, AASCM demonstrated the highest efficacy in withstanding freeze-thaw-chloride ion interactions following the incorporation of coal ash, recycled rubber, and straw fiber, aligning with the mass loss rate law.

### 3.4. Microscopic Analysis

#### SEM Analysis of Freeze-Thaw-Chloride Coupled Specimens

**Figure 22:**
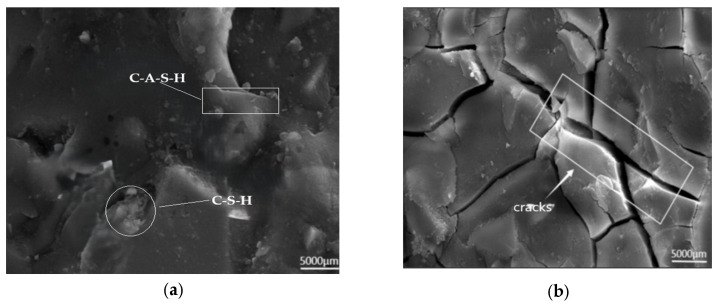
AAS-1 SEM image (5000×), (**a**) AAS-1 conservation 28 d, (**b**) AAS-1 coupling 100 times.

Figure 22 displays SEM images of AAS-1 following 28 days of maintenance (Figure 22a), and 100 cycles of tests coupling freeze-thaw and chloride ions (Figure 22b). Slag hydration generated a greater quantity of C-S-H gels and a lesser amount of C-A-S-H gels. In SEM images, C-S-H is composed of particles with diameters approximately ranging from 5.0 to 6.4 nanometers (nm), and these particles are formed by the stacking of some ellipsoidal basic structures. In contrast, C-A-S-H exhibits more regular crystalline morphologies. The overall structure displays a disrupted and interconnected configuration. AAS-1 has more cracks now that it has been frozen and thawed 100 times with chloride ions present [35].

**Figure 23:**
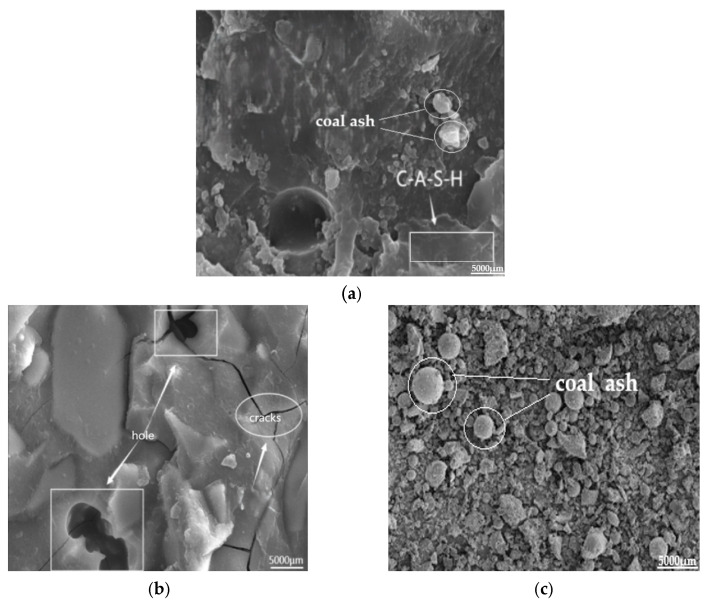
AAS-3 SEM image (5000×). (**a**) AAS-3 conservation 28 d, (**b**) AAS-3 coupling 100 times, (**c**) Microscopic pictures of coal ash.

Figure 23 displays SEM images of AAS-3 following 28 days of maintenance (Figure 23a) and 100 cycles of tests involving freeze-thaw and chloride ion coupling (Figure 23b). The unreacted coal ash particles in AAS-3 filled up the inside of the structure, the microstructure of coal ash is shown in Figure 23c. When the coal ash and slag were mixed with water, denser C-A-S-H gels formed, which caused more cracks and holes after 100 cycles of freeze-thaw-chloride ion coupling [36].

**Figure 24:**
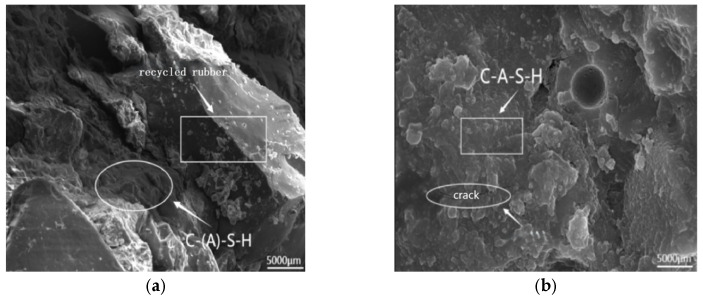
AAS-10 SEM image (5000×). (**a**) AAS-10 conservation 28 d, (**b**) AAS-10 coupling 100 times.

Figure 24 displays SEM images of AAS-10 after 28 days of curing (Figure 24a) and 100 cycles of tests coupling freeze-thaw and chloride ions (Figure 24b). The AASCM with coal ash and recycled rubber works well after 100 cycles of freeze-thaw and chloride ion interaction. The recycled rubber is effectively mixed with C-(A)-S-H (hydrated calcium silicate and hydrated calcium silica-aluminate), which makes the originally brittle material tougher. As a result, the damage caused by the freezing and expansion of free water within the matrix is minimal, leading to only a few cracks [37].

**Figure 25:**
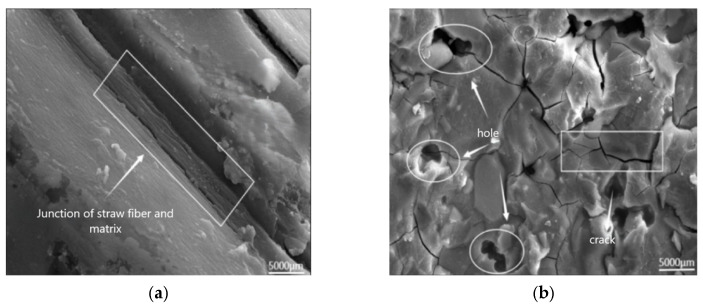
AAS-14 SEM image (5000×). (**a**) AAS-14 conservation 28 d, (**b**) AAS-14 coupling 100 times.

In Figure 25, SEM pictures of AAS-14 are shown after it had been maintained for 28 days (Figure 25a) and had been tested 100 times for freeze-thaw and chloride ion coupling (Figure 25b). After 100 cycles of freeze-thaw-chloride ion coupling, the AASCM incorporating coal ash and straw fibers shows increased damage at the fiber-matrix interface within the specimen, leading to more internal voids. This indicates a predominant freeze-thaw process, which manifests macroscopically as greater damage during the freeze-thaw-chloride ion interaction.

**Figure 26:**
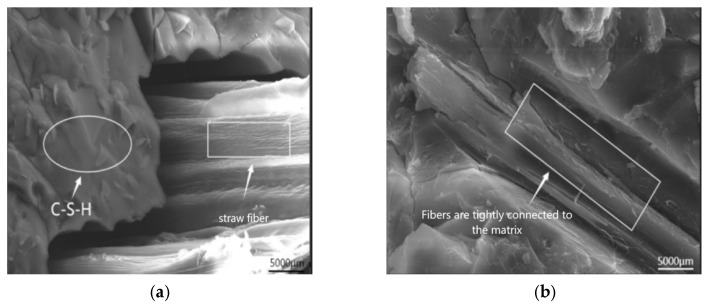
AAS-15 SEM image (5000×). (**a**) AAS-15 conservation 28 d, (**b**) AAS-15 coupling 100 times.

Figure 26 shows SEM images of AAS-15 after 28 days of maintenance (Figure 26a) and 100 cycles of freeze-thaw and chloride ion coupling tests (Figure 26b). The AASCM matrix remains largely unchanged after 100 cycles of freeze-thaw and chloride coupling. The addition of recycled rubber enhances the AASCM’s resistance to frost, while the straw fiber effectively absorbs the incoming chloride ions [38].

## 4. Conclusions

In order to enable AASCM to be applied to buildings and roads in cold regions, this paper conducted experimental research on the combined effects of freeze-thaw cycles and chloride ion corrosion on AASCM with varying ratios. The analysis was performed microscopically using SEM and XRD methods. The study yielded the following conclusions:

(1) During freeze-thaw cycles, AASCM that had both coal ash and recycled rubber lost more mass and less compressive strength than AASCM that was made of only coal ash. The combination made the AASCM more resistant to frost. The addition of coal ash and straw fiber to the AASCM resulted in a decrease in the ion concentration in the deeper layer. This improved it at absorbing chloride ions after 180 days of immersion. The AASCM that had coal ash and recycled rubber added to it was better at absorbing chloride ions than the AASCM that only had one additive.
(2) AASCM, which incorporates coal ash, recycled rubber, and straw fiber, significantly improves the interaction between freeze-thaw and chloride ions. Its mass loss rate and compressive strength loss rate are less favorable than those of other compositions, thus meeting the durability criteria for cold climates.
(3) SEM analysis indicates that the C-A-S-H gel exhibit a comparatively dense and stable structure. The incorporation of recycled rubber into the AASCM matrix can postpone crack formation, thus augmenting the material’s brittleness, while simultaneously improving its ductility and frost resistance. Additionally, the straw fiber in the AASCM matrix acts as a bridging agent, and the C-S-H gel mixes with it in a big way, which helps stop cracks from forming and spreading and also has some ability to absorb chloride ions. After XRD analysis, it was found that AASCM with 20% coal ash makes more C-A-S-H gels, which improves the material’s mechanical properties.
(4) At present, there are still some limitations in the research on the freeze-thaw cycle and chloride corrosion of AASCM, mainly reflected in the lack of systematic comparison of coupled damage behaviors, the lack of in-depth comparative analysis with existing theoretical models, and the limited application of quantitative indicators. In the future, by establishing more comprehensive benchmark tests, including key chloride thresholds, freeze-thaw damage indices, and other parameters, combined with quantitative indicators such as percentage differences and effect sizes, it is expected to more accurately evaluate the durability of AASCM and verify its improvements and innovations in practical applications.

## Figures and Tables

**Table 1:** Chemical composition of slag.

Component	SiO_2_	Al_2_O_3_	CaO	MgO	Fe_2_O_3_	Else
Content (%)	36.9	15.66	37.57	9.3	0.36	0.57

**Table 2:** Chemical composition of fly ash.

Project/Technical Requirements	Grade I	Grade II	Grade III
Fineness (45 μm square hole sieve)	12.0	25.0	45.0
Water demand ratio	95	105	115
Heat loss	5.0	8.0	15.0

**Table 3:** Mix ratio design.

Number	Slag (kg)	Coal Ash (kg)	Recycled Rubber (kg)	Standard Sand (kg)	Straw Fiber(kg)	Water Gel Ratio
AAS-1	1200	/	/	/	/	0.21
AAS-2	1080	120	/	/	/	0.21
AAS-3	960	240	/	/	/	0.21
AAS-4	840	360	/	/	/	0.21
AAS-5	1200	/	60	/	/	0.21
AAS-6	1200	/	120	/	/	0.21
AAS-7	1200	/	180	/	/	0.21
AAS-8	960	240	60	/	/	0.21
AAS-9	900	240	60	60	/	0.21
AAS-10	840	240	60	120	/	0.21
AAS-11	780	240	60	240	/	0.21
AAS-12	1200	/	/	/	12	0.21
AAS-13	960	240	/	/	12	0.21
AAS-14	960	240	/	120	12	0.21
AAS-15	960	240	60	120	12	0.21

Note: C30 is standard concrete with a specific water ratio. Cement, sand, and gravel are in the ratio of 325:185:662:1228. The ratio of cement to standard sand is 0.38:1:1.11, resulting in a water mass of 350 g, a cement mass of 921 g, and a standard sand mass of 1022 g, with a 28-day compressive strength of 34.2 MPa.

## Data Availability

The data presented in this study are available on request from the corresponding author. The data are not publicly available due to privacy reasons.
